# Optimization of cross-border E-commerce (CBEC) supply chain management based on fuzzy logic and auction theory

**DOI:** 10.1038/s41598-024-64123-3

**Published:** 2024-06-18

**Authors:** Zhi Gong

**Affiliations:** Winchester School of Art, University of Southampton, Winchester, UK

**Keywords:** Fuzzy logic, E-commerce, Prediction, Sales forecasting, Computational science, Computer science, Information technology

## Abstract

People have benefited enormously from e-commerce’s explosive expansion in recent years. E-commerce, in contrast to the traditional business environment, is dynamic and complicated, which poses a number of challenges. The prediction market can create mixed intelligence for sales forecasting, which is essential for e-commerce enterprises, to handle this difficulty. Combining the usage of human analysts and machine learning algorithms can accomplish this. To accurately anticipate retailer volume and allot resources, a novel methodology for optimizing supply chain management at CBEC is proposed in this paper. The framework improves efficiency and profitability by using fuzzy logic and auction theory to make strategic decisions. Thanks to this creative strategy, managers can now make more informed decisions, ultimately enhancing the efficiency of CBEC’s supply chain. The results of this paper reveal that our proposed method is superior to previous comparable methods, with RMSE and MAE values of 22.31 and 18.76, respectively. This approach offers a promising solution to the challenges faced by e-commerce businesses, and can help them achieve greater success in the dynamic and complex world of online commerce.

## Introduction

The gathering of a complete sample of data is now possible because to the quick growth of e-commerce, Internet, and big data technologies, and numerous mobile devices. This is because it is now simpler to access the data created or kept by customers when they purchase online. E-commerce businesses may determine the preferences of client groups for various products and forecast the demand and inventory of each product across various time periods by analyzing these data. According to the anticipated outcomes, businesses can assist decision-making for operations, management, and production as well as providing a framework for inventory control, reducing inventory costs for businesses and raising consumer satisfaction with their purchases^1^. Customers can conduct online transactions and make online electronic payments on this platform without actually meeting the merchants. Since 2013, more and more e-commerce businesses have focused on using the Internet to provide customers with high-quality service. Additionally, as online trading grows in acceptance, more people opt to engage in it, creating a significant amount of data about consumer purchasing patterns. As a result, one of the research areas is how to leverage data information to investigate and mine the user behavior law contained in it and use it to projecting commodities sales^2^.

E-commerce businesses, still are encountering previously unheard-of difficulties in producing precise forecasts due to the pervasive dynamic business environments and the rapid technology development requirements. This is due to the fact that consumers’ shopping habits on e-commerce platforms are unique from those of traditional offline retailers and are subject to swift change^3^. Presently, there are two basic methodologies for predicting commodity demand: qualitative prediction and quantitative prediction. The accuracy of qualitative predictions will also vary significantly depending on the predictor’s experience with commodity sales, making them unsuitable for forecasting a wide range of commodities^4^. Simple moving average method and weighted moving average method are two examples of quantitative prediction techniques. Only commodities with reasonably consistent demand and no seasonal trend are eligible for using the weighted moving average approach or the simple moving average method. Additionally, the weights are still assigned for the weighted average technique in a very subjective manner^5^. Over the past few decades, a lot of effort has been put into creating and upgrading forecasting models in order to effectively estimate sales^6^.

Artificial intelligence approaches and traditional linear statistical methods are two categories for existing methodologies^3,7^. The primary components of linear statistical approaches are moving averages, autoregressive integrated moving averages (ARIMA), and other statistical time series analysis models^8,9^. Despite their widespread usage in sales forecasting, linear statistical models cannot represent characteristics present in many actual time-series data, such as nonlinear patterns, asymmetric cycles, and sporadic outlier findings^10^. Additionally, these linear statistical methods are unable to account for fundamental market economic variables like consumer psychology and unquantifiable aspects of business trustworthiness^11^. Consequently, when data exhibit significant volatility or show no discernible trend or periodicity, statistical approaches fail to generate precise forecasts. Expert systems, fuzzy systems, neural network models like feed-forward neural networks (FFNN), convolutional neural networks, recurrent neural networks (RNN), and long short-term memory (LSTM), as well as other hybrid models, are the main artificial intelligence methodologies^7,11^.

In traditional supply chain management, allocation choices in CBEC are frequently based on past sales data. Stockouts may result from this reactive strategy when demand suddenly increases or decreases. Sales can be lost and customer satisfaction can drop as a result of order fulfillment delays. Keeping an excessive amount of inventory on hand can also waste money and resources.

In this paper, a unique AI-based system that supports a proactive CBEC supply chain management strategy is proposed. We overcome the constraints of reactive approaches by utilizing sophisticated analytics techniques. Our system attempts to improve overall supply chain efficiency and optimize resource allocation by combining machine learning, auction theory, and fuzzy logic.

The data-driven theory building principles recommended in^12,13^ and^14^ are consistent with our findings. We not only pinpoint a practical issue with CBEC supply chains, but we also go into greater detail about the "why" of our suggested solution. We further the field’s understanding in this area by concentrating on the causal mechanisms that underlie the performance of our model.

In the context of CBEC, this research suggests a unique AI-based methodology for supply chain management optimization. Our strategy incorporates three essential elements to overcome the shortcomings of current techniques:

- Neural Network-based Demand Forecasting: A custom neural network model predicts future demand for each retailer, considering historical sales data and employing a black-hole optimization algorithm for weight vector adjustment.
- Fuzzy Logic for Resource Needs Assessment: Predicted demand values are combined with current inventory and sales features within a fuzzy model to precisely determine resource renewal needs for each retailer.
- Auction Model for Resource Allocation: An auction model evaluates the value of each seller in terms of resource allocation, ultimately determining the optimal allocation pattern based on these valuations.

The main innovation of our research is this special combination of methods inside the particular framework of CBEC supply chain management, with an emphasis on intelligent resource allocation for various retailers with independent sales platforms. By enabling proactive optimization measures, it improves CBEC profitability and efficiency above typical reactive methods. The following are the main contributions of the paper:

- Offer an auction technique to distribute resources fairly among retailers.
- Using optimization and machine learning techniques to forecast sales at any retailer.
- Outlining a hybrid strategy to improve cross-border e-commerce’s (CBEC) supply chain’s efficiency.
- Using a fuzzy model to predict each seller’s level of needs.

Following is a breakdown of the remaining sections of the paper: We discuss the literature review in “Litereature review”. The suggested system’s technique is described in “Methology”. Section. The results and a comparison of the suggested method are presented in “Simulation and Performance Evaluation”. “Discussion, limitations, and future directions” includes the discussion, limitations, and future works, and finally, the paper is wrapped up in “Conclusion”.

## Literature review

As e-commerce has grown in popularity, a growing number of approaches have been suggested and used to anticipate commodity sales, including the ones we’ll discuss as follows: logistic regression, decision tree, random forest, gradient ascending decision tree, neural network, fuzzy, deep learning and so on.

Singh et al.’s^15^ machine learning methods for predicting e-commerce platform sales were presented. The model with the best prediction range, where the anticipated value and the actual value are practically similar, was selected as the best method by the authors after they were able to construct and test all of the machine learning models they had chosen. Pan et al.^16^ mined e-commerce data with a convolutional neural network to forecast product sales. It combines pertinent product data with shipping log data, extracts useful features, and then makes use of these qualities to forecast sales. The technique’s validity and effectiveness in predicting sales are confirmed using actual e-commerce data sets. Li et al.^17^ forecasted weekly sales using the ECS-ARIMA model utilizing Jingdong Company’s e-commerce sales data. The model had trouble fitting nonlinear patterns with significant local errors but did well with linear patterns.

Stripling et al.^18^ employed the logistic regression approach to explore client classification challenges in the telecommunications business in order to prevent customer loss. Bell et al.^19^ utilized a decision tree to forecast customer purchasing behavior. When compared to other prediction approaches, it can provide logical classification in a clear and intuitive manner. Mahdavinejad et al.^20^ successfully employed a perceptron vector machine, logistic regression, and random forest fusion model to the project of anticipating customers’ repeated purchase behaviours on an e-commerce platform.

Liberis et al.^21^ employed a convolutional neural network approach to create a model to estimate the possibility that customers would make repeat purchases, and found that the convolutional neural network method had a sizable capacity for learning for characteristic variables with intricate non-linear correlations. Pham et al.^22^ applied a convolutional neural network to encode the image of each commodity as real number vectors, and then solved nonlinear optimization problems based on these real number vectors to create commodity prediction information. Semantic analysis of user-posted content was used to determine the topic content and entity type used by Khaled et al.^23^. Then, for semantic improvement to show user behavior, they gathered more content connected to the topic from related other websites. Finally, they developed a model to deliver user-specific recommendations. Madhuvanthi et al.^24^ showed how to use a machine learning algorithm to project automobile sales. This study focuses on the information on car sales and how it is acquired from diverse sources. The main issues that the researcher discovered were identifying differing viewpoints on how effectively the various criteria in our dataset perform and selecting the optimal algorithm to use.

Bohanec et al.^25^ examined the explanation of machine learning models in sales forecasting. This study primarily examines the key machine learning models that are frequently applied to forecasting sales, and it also analyses the top model currently in use. The issue that this study paper identified was how to choose the proper model based on the understanding of the business through the use of intelligence and data driven models. A different investigation was carried out by Yu et al.^26^ to examine the sales projection for Amazon sales based on several statistical methodologies. The majority of this study’s attention was given to Amazon data, which was used to anticipate future sales using previous data and statistical algorithms. How a statistical sales approach can aid in sales forecasting is the issue that the instructor in this research has recognized. Neelakandan et al.^27^ proposed a methodology for forecasting online product sales using a continuous stochastic fractal search (SFS) technique. They use a time series dataset to evaluate the performance of a deep learning-modified neural network (DLMNN). The study shows that the unsupervised pertained DLMNN model outperforms the non-deep learning model in predicting sales, with improved RMSE, mean, and standard deviation.

### Impact of COVID-19 on CBEC supply chains

Global supply chains were severely disrupted by the COVID-19 epidemic, and CBEC, faced particular difficulties. Production and distribution of commodities were disrupted as a result of workforce shortages and delays in transportation brought on by lockdowns and travel restrictions^28^. Due to a shortage of inventory, e-commerce companies found it difficult to keep up with the rapid growth in online demand. These problems were made worse by border closures and tighter customs laws, which emphasizes the need for a more comprehensive approach to information management in supply chain^29^.

The necessity for more resilient and flexible supply chain management techniques in CBEC is highlighted by these interruptions. Our suggested methodology seeks to enhance resilience in multiple ways by utilizing cutting-edge analytics methods including fuzzy logic, auction theory, and machine learning.

- Better Demand Forecasting: Our model can produce more accurate demand projections by using historical sales data and taking into account external influences. This helps firms better anticipate needs and optimize inventory levels.
- Proactive Resource Allocation: A proactive strategy replaces the previous reactive one in the model. The danger of stockouts can be reduced by proactively allocating resources to fulfill projected demands by forecasting future demand and seller performance.
- Improved Supply Chain Visibility and Collaboration: Improved supply chain visibility is made possible by the integration of several data sources. Better cooperation between e-commerce companies and their foreign partners is made possible by this, giving them a greater ability to respond quickly to interruptions.

## Methodology

In this research, a new framework is proposed to improve supply chain management in CBEC, combining optimization techniques and machine learning for sales volume prediction of factors, and using fuzzy logic and auction theory to allocate resources to sellers. This section presents the suggested design after detailing the data structure that was employed in the study.

### Data

The research data was collected over a two-year period through the sales records of 10 active sellers in the CBEC domain. All sales factors are supported through a common supplier, and each seller utilizes multiple online sales platforms for marketing and selling their products. The collected data in this research describes the weekly sales volume for each seller and includes only records of physical product sales. The number of online sales platforms used by each seller ranges from 3 to 7, and some factors use shared platforms for product sales. The data used in the current study consists of 110 records for each seller, describing the total sales volume in the last seven days (across all platforms used) as a natural number within the range of 44 to 185. In addition to sales volume, each data record includes a set of features appears in Table 1.

**Table 1:** Features of each database record.

Indicator	Title	Description	Type
I_1_	Week	Week of the month	Numeral
I_2_	Month	Month of the year	Numeral
I_3_	Order Volume	Number of orders in the past week	Numeral
I_4_	Retailer Stock	Current inventory of retailer in warehouse	Numeral
I_5_	Total Value	Total value of orders in the past week	Numeral
I_6_-I_12_	Order History	History of orders in the last 7 weeks as a numeric vector with length 7	Numeral
I_13_-I_19_	Delay History	History of retailer’s delay in fulfillment of orders in the last 7 weeks as a numeric vector with length 7	Numeral

Thus, each data record is described by 19 features that are used in the process of predicting the future sales volume of each retail seller.

### Proposed framework

In the proposed framework for optimizing supply chain management in the CBEC system, it involves a set of retail sellers, each utilizing multiple online sales platforms for marketing and selling their products. Each retail seller has a warehouse to store their resources, which is supported by a common supplier. In traditional supply chain models, online customers place orders through the desired product sales platforms, and the retail seller fulfills the customer’s order using their warehouse inventory. When the inventory of a product decreases or runs out, the retail seller will request support from the common supplier. This reactive process in supply chain management can lead to decreased efficiency in the CBEC sales system because delayed product supply by the supplier can result in customer dissatisfaction and attrition.

On the other hand, maintaining excess product inventory in the retail sellers’ warehouses can lead to resource wastage in the supply chain. Additionally, supplying a common product (with limited inventory) among multiple retail sellers needs to align with each seller’s performance. These characteristics make it necessary for the supply chain system to have an active strategy for fulfilling the demands effectively.

**Figure 1:**
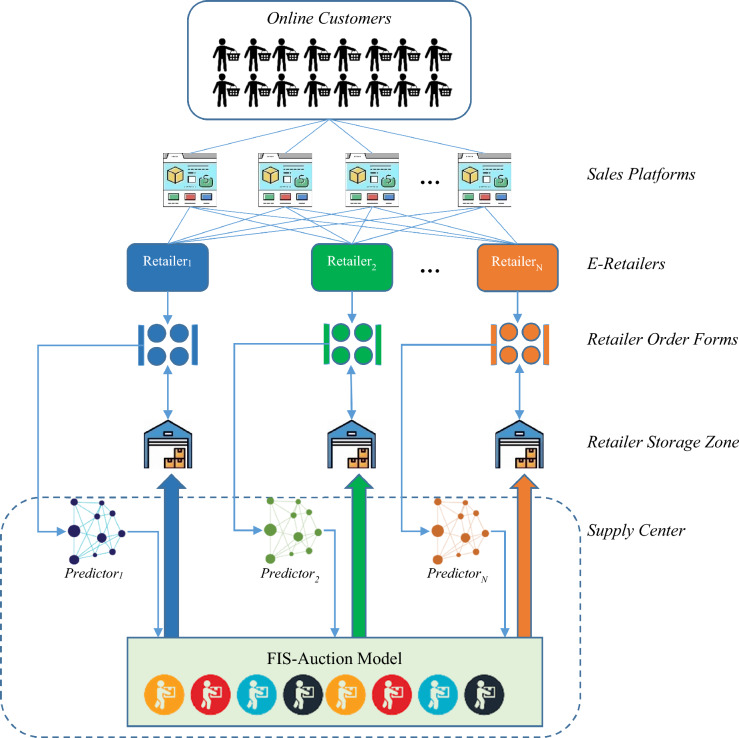
Proposed Framework for Supply Chain Performance Optimization.

In this paper, a combined strategy for optimizing supply chain performance in CBEC is presented, which fairly distributes limited supplier resources among retail sellers while considering the forecasted needs of the retail sellers. The mechanism of the proposed strategy is depicted in Fig. 1.

Figure 1 demonstrates, the proposed method focuses on intelligent resource allocation for retail sellers in a CBEC system. The suggested framework is made up of three primary parts:

- A prediction model to estimate the future orders of each retail seller.
- A fuzzy model for evaluating each seller’s resource requirements.
- An auction model for the fair distribution of resources among retail sellers.

In this approach, the future needs of each retail seller are predicted using a custom neural network-based learning model. This learning model, trained based on the sales history of the respective seller, utilizes a black-hole optimization algorithm to adjust its weight vector. The prediction values obtained from this neural network are then used to determine the seller’s resource requirements through a fuzzy model.

**Figure 2:**
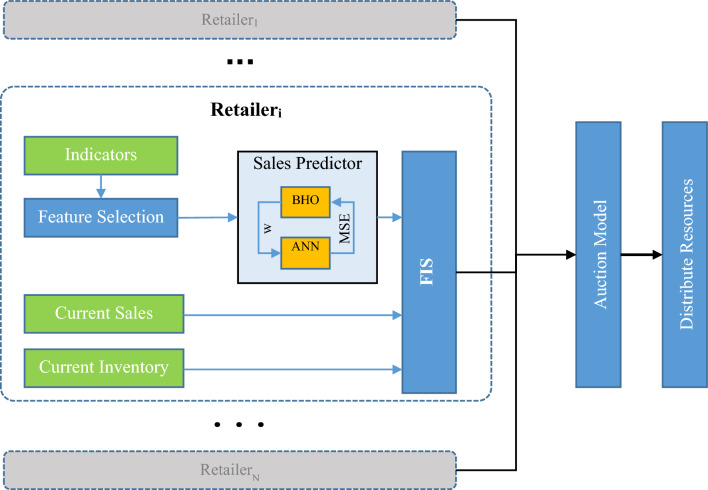
Determining the Resource Allocation Pattern among Retailers in the Proposed Methodology.

After executing the above processes for all retail sellers, the value of each seller in terms of resource allocation is estimated using an auction model. Ultimately, the resource allocation pattern is determined based on the values assigned by this auction model. The proposed resource allocation model for retail sellers’ mechanism is depicted in a diagram in Fig. 2.

As shown in Fig. 2, the proposed model, after selecting relevant indicators for the order volume of each retail seller, uses a separate ANN (Artificial Neural Network) for predicting the future order volume for that seller. This ANN model utilizes a black-hole optimization algorithm to determine its weight and bias vector, aiming to find a combination of weight values that minimizes the training error. Using an independent ANN model for each retail seller allows for a more precise modeling of each seller’s transaction patterns. This approach can better identify functional differences among sellers in terms of marketing and sales in different time periods.

The predicted values from the ANN model, along with the current inventory and current sales features, are used as inputs to a fuzzy model to estimate the retail seller’s resource renewal needs for the next period as a fuzzy variable. In Fig. 2, the details of these processes for retail seller i are depicted.

The processes of predicting future sales and assessing the resource renewal needs for each retail seller are repeated, and the results from these processes are used as inputs to an auction model. This auction model describes the value of each seller for resource allocation as a numerical value. Finally, by normalizing these values, the resource allocation pattern among retail sellers is determined.

The continuation of this section provides a detailed explanation of each of the above-mentioned stages.

#### Sales volume prediction based on ANN and BHO

The first step in the proposed method involves predicting the future sales volume of retail sellers using optimization and machine learning techniques. Accurate sales volume prediction can help estimate the resource needs of sellers for future periods and manage the supply chain accordingly. Since each seller has a different performance pattern, and their sales volume is directly related to their marketing activities, it’s not feasible to use a shared model for predicting the sales of all sellers. For this reason, in the proposed method, each retail seller utilizes a separate learning model to predict their sales volume, which is trained solely based on features related to their performance history.

However, to achieve an efficient prediction model, two fundamental issues need to be addressed. Firstly, sales volume prediction should be based on indicators related to the seller’s performance pattern. For example, for a seller who intensifies their marketing activities during specific time intervals, time-based indicators are more important compared to sellers whose marketing activities are consistent over time. The second issue is the necessity of using an optimized configuration for the learning model to ensure the minimum prediction error. The proposed model tackles the first issue using a feature selection strategy based on ANOVA and FSFS (Feature Selection via Feature Space). To address the second requirement, the model uses the BHO (Black-Hole Optimization) algorithm to fine-tune the ANN model. Consequently, the sales volume prediction process in the proposed method consists of two detailed steps, which are discussed further in this section.

(A) Feature Selection Before training the ANN model based on features related to the performance history of the retail seller, a feature selection algorithm is used to determine the most relevant factors with the sales pattern. Removing irrelevant factors can not only increase the processing speed of the model but also have an impact on reducing its error while reducing the model’s dependence on multiple data points. These advantages make the feature selection process highly important for achieving an efficient prediction model. The suggested technique does this by combining the FSFS algorithm and ANOVA.

In this process, each data record is first described as follows:

1 $${Y}^{t+1}:\langle {I}_{1}^{t},{I}_{2}^{t},\dots ,{I}_{19}^{t}\rangle$$

where I_k^t represents the k-th feature describing the performance of the retail seller in a time period (week) t, which is extracted based on Table 1. Additionally, Y^(t + 1) represents the sales volume in the next week (t + 1), which can be extracted from the next data record related to sales data.

In the feature selection algorithm used, all candidate factors (Eq. 1) are first ranked using one-sided ANOVA analysis to assess the importance of each factor using the F-statistic. The F-value in one-sided variance analysis represents the ratio of between-group variation to within-group variation, where higher F-values indicate that the data between groups is more diverse compared to within groups. This property can indicate the presence of a meaningful and different pattern for identifying data groups.

Existing features are rated in descending order according to their F-statistic values in order to choose relevant features for the proposed technique. Then, the SFS (Sequential Forward Selection) algorithm is used to identify the right amount of features. In this procedure, the target variable (future sales) is estimated using an artificial neural network-based estimating model based on various combinations of rated features. In this instance, the target variable is estimated using the two features that had the greatest rankings, and the training error is assessed. Then, the next feature is added to the selected feature set, and the training error is evaluated again. If the training error decreases compared to the previous iteration, the added feature is retained; otherwise, the feature is removed from the selected feature set, and the process ends.

Finally, a combination of ranked features that results in the lowest training error is considered as the selected feature set. It’s important to note that the feature selection process is carried out separately for each retail seller.

(b) Sales Volume Forecasting.

After determining the most relevant indices, the process of predicting future sales volume based on these indices is carried out. If we represent the selected set of indices by S = {I_(s(1))^t, I_(s(2))^t, …, I_(s(n))^t}, then the prediction process can be formulated as follows:"

2 $${Y}^{t+1}=f\left(S\right)=f({I}_{s(1)}^{t},{I}_{s(2)}^{t},\dots ,{I}_{s(n)}^{t})$$

In the above equation, "f" displays a function that models the relationship between performance factors at time "t" and the sales volume at time "t + 1". An ANN (Artificial Neural Network) model is employed in this step of the suggested methodology to estimate this relationship. Within the proposed approach, the sales volume prediction model for each retailer is created using an Artificial Neural Network.

**Figure 3:**
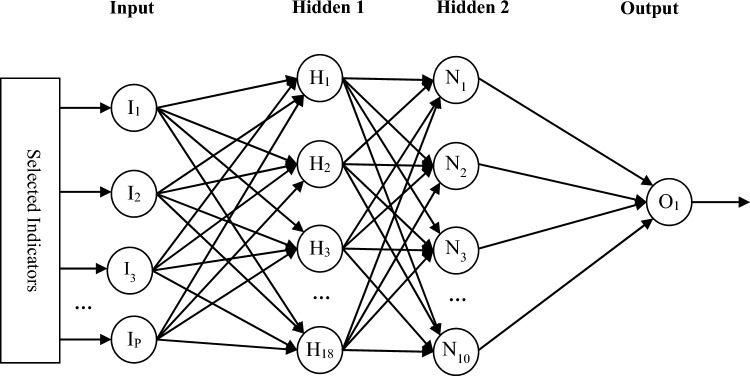
The Structure of the Neural Network Used in the Proposed Retailer Sales Volume Forecasting Method.

The BHO (Black Hole Optimization) approach is used to modify the neural network’s weight vector as opposed to more conventional training procedures. The goal of this optimization procedure is to establish the neural network’s weight vector based on the training error (which serves as its own fitness function). Figure 3 depicts the structure of the neural network utilized in the suggested technique for forecasting each retailer’s sales volume.

In Fig. 3, the neural network structure used in the proposed method for predicting the sales volume of each retailer is depicted. The neural network consists of an input layer, two hidden layers, and an output layer. The input layer is fed with the selected features from the previous step. The first and second hidden layers contain 18 and 10 neurons, respectively, with sigmoid logarithmic and linear activation functions assigned to these two layers. Finally, the output layer comprises a single neuron that represents the predicted sales volume for the input sample.

As mentioned, the BHO (Black Hole Optimization) algorithm is used for training this neural network. In the following sections, the structure of the response vector and the fitness function for convergence evaluation will be explained, followed by the steps of training the ANN model using BHO.

The neural network’s biases and the weights of connections between neurons are determined by the response vector in the BHO algorithm, which is employed in the proposed method. The BHO algorithm’s response vector length for a neural network with I input neurons, H1 neurons in the first hidden layer, H2 neurons in the second hidden layer, and P output neurons will be equal to:

3 $$L={H}_{1}\times \left(I+1\right)+{H}_{2}\times \left({H}_{1}+1\right)+P\times ({H}_{2}+1)$$

In the equation above, H1 × (I + 1) represents the number of weight values between the input layer and the first hidden layer plus the bias of the first hidden layer. H2 × (H1 + 1) indicates the number of weights between the first and second hidden layers plus the bias of the second hidden layer. Finally, P × (H2 + 1) signifies the number of weights between the last two layers plus the bias of the output layer. Thus, the length of each solution vector in BHO will be L. In this vector, the weight and bias values are described as real numbers within the range [-1, + 1]. In other words, each optimization variable in BHO is represented as a real variable with search bounds [-1, + 1].

Using a fitness function, the BHO algorithm assesses the fitness of each solution. The outputs of the neural network are generated for the training samples and contrasted with the actual goal values once each response vector’s weights have been determined. The effectiveness of the neural network and the calibre of the response generated are then assessed using the mean squared error (MSE). As a result, the following definition will serve as the fitness function for the BHO algorithm:

4 $$MSE=\frac{1}{N}\sum_{i=1}^{N}{\left({y}_{i}-{t}_{i}\right)}^{2}$$

In the equation above, N stands for the quantity of training samples, ti for the i-th training sample’s goal value, and yi for the neural network’s output for the i-th training sample. In the suggested approach, a neural network topology that can reduce the fitness function is chosen using an optimisation technique.

It’s important to note that the BHO algorithm randomly generates the initial population.

*Step 1* Make a random initial population of solution vectors in step 1.

*Step 2* Apply Eq. (4) to get the fitness for each solution vector.

*Step 3* Define the black hole (X_BH) as the solution with the lowest fitness.

*Step 4* Reposition each answer (star) so that it is similar to X_i as follows:

5 $${X}_{i}={X}_{i}+rand.({X}_{BH}-{X}_{i})$$

In Eq. (5), X_i denotes the location of star/solution i in the problem space, while X_BH denotes the location of the black hole. The term "rand" refers to a random number between 0 and 1.

*Step 5* Perform the following calculation to determine the distance at which a star can be sucked up by a black hole:

6 $$R=\frac{fitness(BH)}{\sum_{i=1}^{N}fitness(i)}$$

N here refers to the quantity of solution vectors used in the BHO algorithm.

*Step 6* Replace the existing black hole with a solution vector if its fitness is lower than the black hole’s.

*Step 7* Substitute a new random solution vector for any solution vectors that are closer to the black hole than the threshold R.

*Step 8* Continue to the next step if the number of algorithm iterations surpasses the threshold T; else, start over at step 2 with the search.

*Step 9* Present the black hole with the lowest fitness as the most effective solution that has been found.

The ANN model produced by using this solution vector will be employed by the store for sales volume forecasting in subsequent time intervals when the best solution has been determined based on the aforementioned processes.

#### Evaluating each retailer’s resource requirements using fuzzy logic

**Figure 4:**
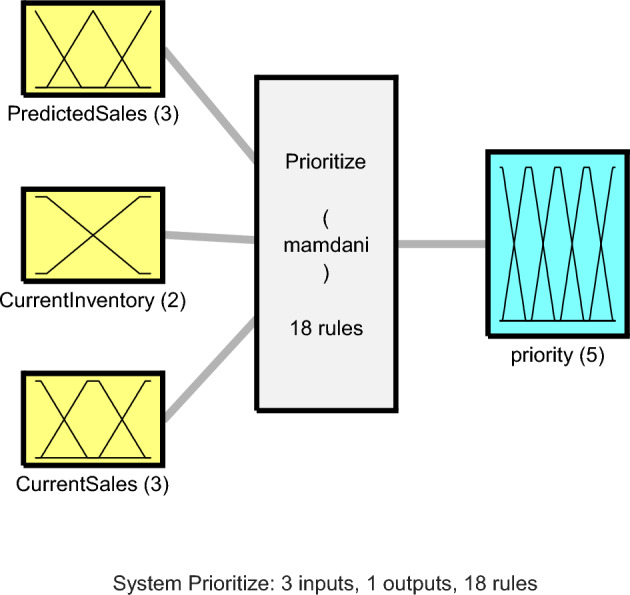
Proposed Fuzzy Model for Assessing the Retailer’s Resource Renewal Level.

The suggested technique’s second step, a fuzzy logic model is used to assess the resource requirements of each retailer. Using fuzzy logic for this task allows modeling decision-making under uncertainty, which increases the flexibility of the model for decision-making in the next step. This fuzzy logic model evaluates the resource needs of each retailer based on their current information and predicted status. The structure of this fuzzy model is depicted in Fig. 4.

**Figure 5:**
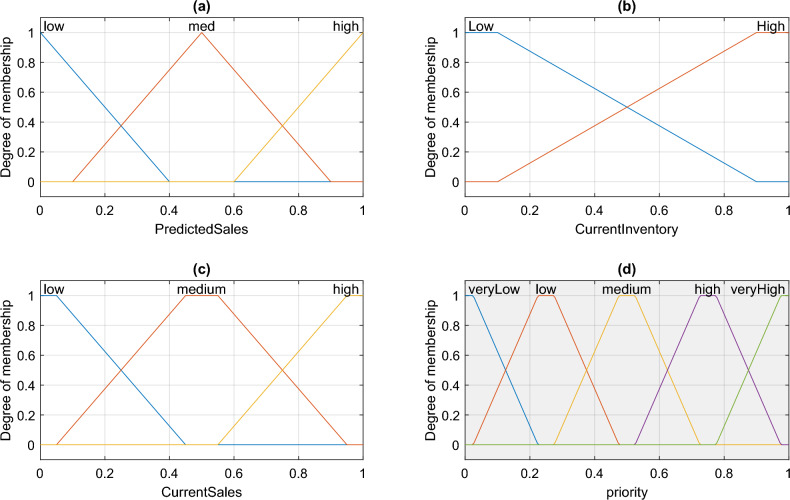
Membership Functions of Fuzzy Variables in the Proposed Method.

The fuzzy model utilised in Fig. 4 is the Mamdani fuzzy inference system, which contains 18 fuzzy rules, one output variable, and three input variables. The membership functions for the input and output variables in this fuzzy system are shown in Fig. 5.

In Fig. 5a, the membership functions of the predicted sales volume, which is essentially the value predicted by the neural network in the previous step, are displayed. Figures b and c show the membership functions for the current sales volume and inventory level, respectively, which are the input variables. Finally, the output fuzzy variable with 5 membership functions is presented in Figure d. In the following, I will explain how each of these variables is calculated.

The expected sales volume for the forthcoming period is the first input of this fuzzy system and is determined by the proposed neural network and described by three membership functions: "low," "medium," and "high." This input variable’s normalization for the suggested fuzzy model is as follows:

7 $${I}_{1}=\frac{Sale{s}_{predict}}{\underset{j}{\text{max}}Sale{s}_{j}}$$

In the above equation, Sales_predict represents the predicted sales volume by the proposed ANN model, and Sales represents the vector of historical sales volume for the current retailer. The retailer’s current inventory level is defined by the second fuzzy input variable, which has the membership functions "low" and "high," and is described by the following equation:

8 $${I}_{2}=1-\frac{Sale{s}_{current}}{Inv}$$

In the above equation, Inv represents the current inventory level of resources in the dedicated store for the current retailer, and Sales_current represents the current sales volume in the backlog. Finally, the third fuzzy input variable in the proposed fuzzy inference system is the current product sales volume, which is described by three membership functions: "low," "medium," and "high," and is calculated using the following equation:

9 $${I}_{3}=\frac{Sale{s}_{current}}{\underset{j}{\text{max}}Sale{s}_{j}}$$

The output of the fuzzy system will use the stated fuzzy variables to determine the degree to which a retailer needs resource replenishment from the supplier. In the proposed fuzzy model, this output variable is determined using the rules provided in Table 2.

**Table 2:** Fuzzy Rules for Determining the Retailer’s Level of Need for Resource Replenishment by the Supplier.

Rule No	Predicted Sales	Inventory	Current Sales	Output
1	Low	Low	Low	Low
2	Low	Low	Medium	Medium
3	Low	Low	High	Medium
4	Low	High	Low	Very low
5	Low	High	Medium	Low
6	Low	High	High	Low
7	Medium	Low	Low	Medium
8	Medium	Low	Medium	Medium
9	Medium	Low	High	High
10	Medium	High	Low	Low
11	Medium	High	Medium	Medium
12	Medium	High	High	High
13	High	Low	Low	High
14	High	Low	Medium	Very high
15	High	Low	High	Very high
16	High	High	Low	Medium
17	High	High	Medium	Medium
18	High	High	High	High

After prioritizing the determination of each retailer’s resource replenishment needs by the supplier, all the calculated results for the retailers are used as inputs to the proposed auction model. Ultimately, the resource allocation to each retailer is carried out through this model.

#### Equitable distribution of resources among small retailers based on auction theory

In the third phase of the proposed method, the allocation of resources to retailers is based on auction theory. Given the predictions of future sales volume and the assessed resource replenishment needs of each retailer, the goal is to distribute resources fairly while maximizing the overall profitability of the CBEC model, considering the constraints of the supplier’s resources.

The theory of auctions, a branch of applied economics, focuses on how individuals behave in auction markets and analyzes the characteristics of auction markets. Over time, various auction mechanisms, efficiency, desirable pricing strategies, optimization, equilibrium of bidding strategies, and income comparisons have been studied extensively by theorists for auctions and related topics. Auctions are transactions where, based on participants’ bids, a particular set of rules and resource allocations are determined. Auctions are classified as incomplete information games because, in most cases, one party does not have complete information about the other party’s deal. For example, participants usually know their own valuation of the product, but this valuation is unknown to other participants and sellers. Auctions come in various forms, but a common feature among them is their universality, as they can be used for the purchase or sale of various items. These characteristics make auction theory a suitable choice for addressing the research problem in this study.

There are many categories for different types of auctions in this theory. For example, auctions can be one-dimensional or multi-dimensional, they can be one-sided or two-sided, bids can be open or sealed, and the items can be single-unit or multiple-unit, and so on. In the proposed method, a sealed-bid auction model with a first-price mechanism is used to determine the resource allocation pattern among the retailers by the supplier. In the proposed method, a sealed-bid auction model with a first-price mechanism is used to determine the resource allocation pattern among the retailers by the supplier. In this auction model, buyers submit their sealed bids simultaneously to the sellers. These bids are observed by the sellers, and the most acceptable price will be chosen. In this auction model, no one will be able to see the rival’s bid before submitting their ownIn the proposed model, the retailers in need of resources are considered as buyers in the sealed-bid auction model, and the common supplier in the supply chain is considered as the seller of this model. The item being traded, in reality, represents the resources to meet the needs of the retailers. In this case, the price is also described as a function of the current status and the future status of the seller.

In the proposed auction model, a time period, such as "s," is considered, representing the time interval that covers the resource provisioning for the retailers. In each execution of the proposed auction model, the supplier offers resources to the buyers for the time period "s." Participants in the auction include the set of previously unsuccessful retailers from the previous auction and the new retailers in need of resources.

The goal of the proposed auction model is to distribute "m" products belonging to the supplier among "n" products needed by the retailers. If "n" is greater than "m," at least one retailer will fail in the auction process and will need to participate in the next auction to secure the required resources. In the proposed method, the proposed price for each retailer in the auction model is calculated as follows:

10 $$P=F\times \frac{Sale{s}_{current}+Sale{s}_{predict}-Inventory}{{\text{log}}_{2}(N)}$$

In the above equation:

- $Sale{s}_{current}$ represents the current sales volume awaiting order completion.
- $Sale{s}_{predict}$ describes the predicted sales volume for the time period s + 1 by the ANN model.
- Inventory characterizes the current inventory of the retailer.
- N shows how many times the retailer has been unsuccessful throughout the bidding process.
- F represents the priority value determined by the proposed fuzzy model.

To distribute resources based on the above equation, initially, the supplier removes all retailers with negative proposed prices. The eliminated retailers are considered as unsuccessful buyers in the current auction. For this set of unsuccessful retailers, the value of **N** for each of them is increased by one to improve their chances of success in the next auction. Then, the remaining set of proposed prices (positive prices) is normalized using the following formula.

11 $$P_{i} = \frac{{P_{i} }}{{\mathop \sum \nolimits_{j = 1}^{R} P_{j} }}$$

In the equation above:

- P_*i*_ represents the proposed price by retailer i, which is calculated using Eq. (10).
- R denotes the number of successful retailers in the current auction.

Finally, the allocated share for each retailer is determined using the provided formula.

12 $${Z}_{i}={P}_{i}\times m$$

In the equation above, **m** represents the number of resources available for allocation to all the retailers in the current auction by the supplier.

## Simulation and performance evaluation

The proposed model is put into practice in the present research using the MATLAB software. The model is then tested using the data that is present in the database. Using a tenfold cross-validation technique, the results are made more accurate and reliable. In this scenario, the experiments are run ten times, with the database samples being split into training (90%) and testing (10%) subsets for each run. As this paper was about sales prediction, the model we presented is a machine learning and optimization-based solution that predicts the level of demand for each seller using a fuzzy model. And other models that were used for comparison, respectively, were proposed (All Indicators), ANN (trainIm), SARIMA, CNN and ARIMA-NARNN.

- Proposed (All Indicators): denotes that we have abandoned feature selection in our proposed method.
- ANN (trainIm): indicates that the neural network training conducted using BHO has been disregarded.
- SARIMA: Singh et al.^15^ developed machine learning algorithms for predicting e-commerce platform sales, choosing the model with the area of prediction where actual and anticipated values are most similar.
- CNN: Pan et al.^16^ used a convolutional neural network to predict product sales using e-commerce data, confirming its effectiveness using actual data sets.
- ARIMA-NARNN: Li et al.^17^ used the ECS-ARIMA model to forecast weekly sales using Jingdong Company’s e-commerce data, despite issues with nonlinear patterns and local errors.

**Figure 6:**
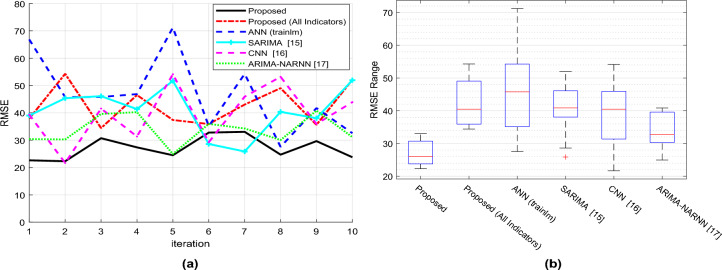
Comparison of prediction results RMSE.

According to Fig. 6a,b, the desirable condition for this metric is decreased prediction error, which is validated by analyzing fluctuations in RMSE and boxplots. In comparison to previous circumstances, the suggested strategy consistently produces reduced squared error and provides higher accuracy with lower RMSE. Due to the lower degree of prediction error and narrower range of error fluctuations, these results point to a higher possibility of correct outputs from the suggested method. The results show that the suggested method is superior to the other methods compared in this study in terms of reliability and accuracy.

**Figure 7:**
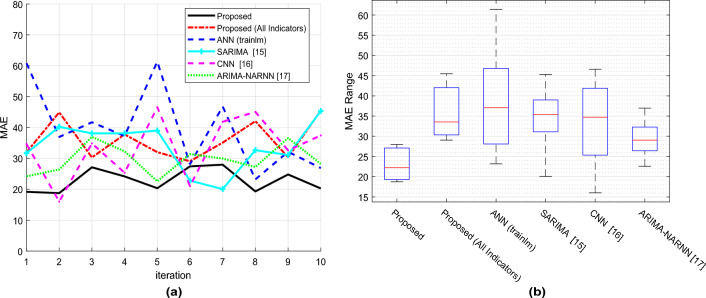
Comparison of prediction results MAE.

Figure 7a displays the Mean Absolute Error (MAE) values at each iteration, indicating that the proposed method can predict the target variable with a lower MAE. Additionally, the suggested method has narrower absolute error variances across multiple iterations, which is favorable for algorithm reliability, as shown by the figure. The box plot of MAE after 10 iterations is shown in Fig. 7b, and each box’s solid line represents the top and lower boundaries of the fluctuations in the algorithm’s absolute error throughout various iterations. The proposed method has a smaller MAE and a smaller range of fluctuations in absolute error than other methods. Additionally, compared to other ways, the suggested method has a greater average accuracy. The smaller range of absolute error variations in the proposed method indicates higher reliability and the suggested method has lower error compared to other methods.

Figures 6a,b and Figs. 7a,b make this clear: when compared to the compared algorithms, the suggested framework consistently shows lower RMSE and MAE values. This shows that the average gap between each retailer’s actual and expected demand levels is less. Put more simply, our model predicts demand numbers that are, generally speaking, closer to the real levels observed. The boxplots provide additional evidence of the suggested method’s smaller range of error variations. As a result, the model’s forecasts will be more consistently made and will be less likely to significantly deviate from real demand.

With a focus on neural network optimization, the suggested framework’s special combination of methodologies yields excellent demand forecasting performance. By capturing complex non-linear relationships in previous sales data, the neural network architecture improves the accuracy of demand forecasts. To further improve prediction accuracy, BHO makes sure the network converges to an ideal weight vector.

**Figure 8:**
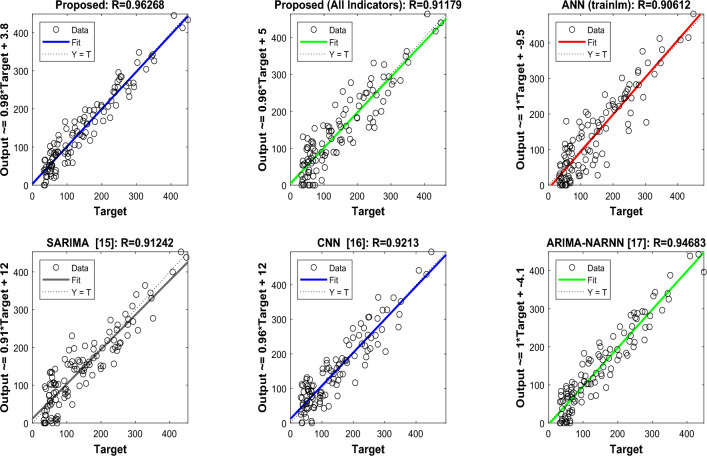
Liner regression plot for the estimated volume of sales model.

Figure 8 in this paper illustrates the projected values for each approach on the vertical axis and compares them with the intended variable. We can state with confidence that our suggested model outperforms the comparison algorithms in terms of sales prediction accuracy after examining the regression graphs in Fig. 8. Our model’s stronger correlation coefficient (R = 0.9627) and centered distribution of regression points show that the anticipated and actual sales figures fit together better. Furthermore, the linear regression equation’s closer slope to 1 denotes a more proportionate relationship between expected and actual sales. These observations can be attributed to the novel combination of techniques used in the model, which includes the incentive design of the auction process, the learning capability of the neural network, and the handling of ambiguity by fuzzy logic. Together, these elements improve the accuracy of sales forecasts and offer useful information to CBEC e-commerce companies.

**Figure 9:**
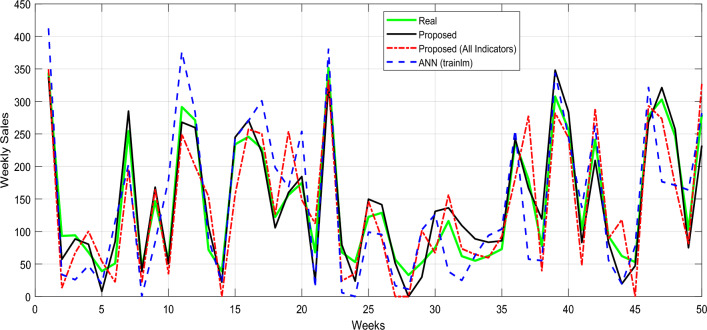
Real values and proposed model predictive values.

The findings shown in Fig. 9 show that the suggested method outperforms the other evaluated algorithms in terms of predicting average sales values. The suggested method’s anticipated values are considerably more in line with the actual values, and the pattern of variations in average sales is also more consistent with the proposed method’s predicted values. This shows that the suggested method’s strategy of choosing pertinent elements and applying weighted averaging can produce outputs with reduced error values and better coverage of projected algorithm errors. These findings demonstrate the value of adopting cutting-edge methods for data analysis and prediction, especially in dynamic and complicated systems like sales forecasting.

**Figure 10:**
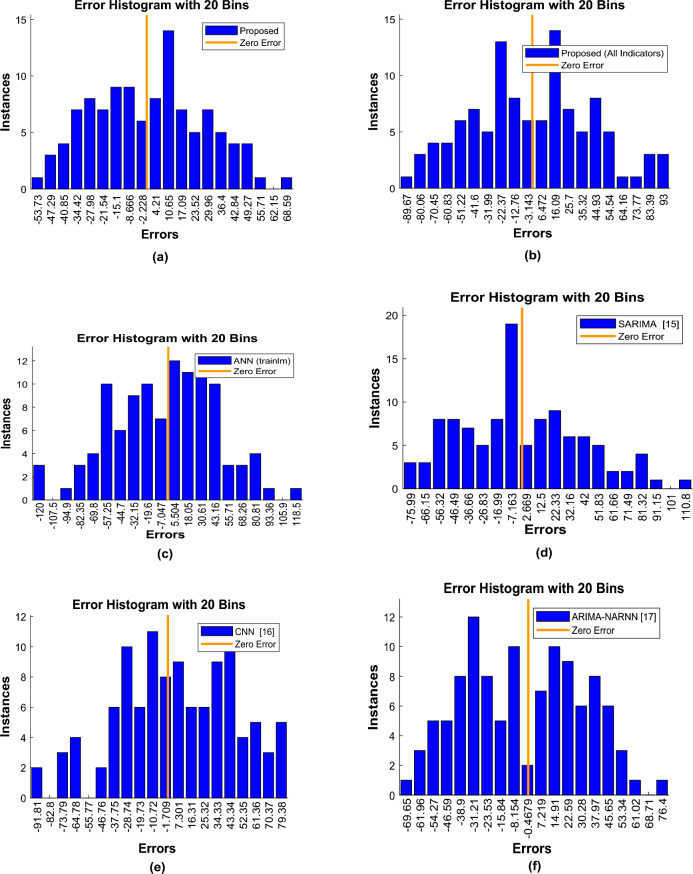
Error histograms.

Examining the error histogram intervals provides a more comprehensive view of the performance of prediction algorithms. The error histogram plots of various sales forecasting techniques for merchants are shown in Fig. 10.

The vertical axis of the graphs in Fig. 10, reflects the frequency of predictions falling within each appropriate interval, whereas the horizontal axis in these plots represents the error intervals in predicting sales. These figures demonstrate that the suggested method’s error interval is smaller than that of the compared approaches. The proposed method’s error boundaries are [− 53.73, + 68.59]. This indicates that, in the worst-case situation, the suggested strategy accurately forecasts the target variable with a 4.5-grade error, which is small compared to other possibilities. Table 3 presents a comparison of different algorithms for predicting average sales values. The algorithms compared include the proposed method, the proposed method with all indicators, ANN (trainIm), SARIMA, CNN, and ARIMA-NARNN. The evaluation metrics used to compare the algorithms are RMSE, MAE, and accuracy. The proposed method outperforms all other algorithms in terms of RMSE and MAE, with values of 27.1563 and 22.9316, respectively. This suggests that the proposed method is better at predicting average sales values with lower error values. The proposed method also has the highest accuracy of 94.8911%, indicating that it is more accurate in predicting the actual values.

**Table 3:** The effectiveness of proposed model and other comparative methods.

Methods	RMSE	MAE	Accuracy (%)
Proposed	27.1563	22.9316	94.8911
Proposed (All Indicators)	42.6296	35.9033	92.0011
ANN (trainIm)	46.7774	39.5172	91.1959
SARIMA^15^	40.8467	33.9089	92.4454
CNN^16^	39.6332	33.5360	92.5285
ARIMA-NARNN^17^	33.7804	29.5854	93.4086

**Figure 11:**
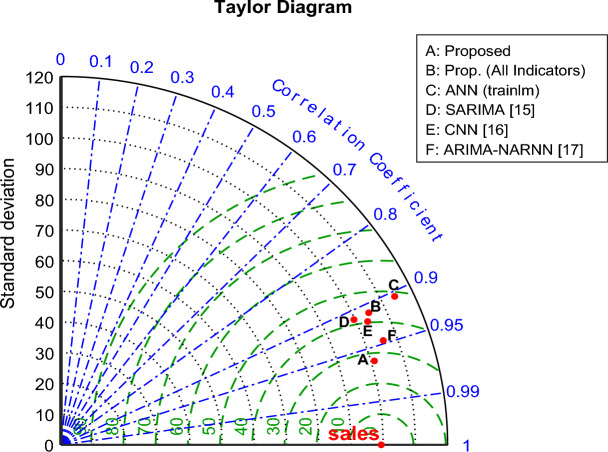
Taylor diagram showing the performance of the evaluated models.

The performance of our suggested framework is plotted on the Taylor diagram with other algorithms in Fig. 11. The ideal approach would be one that lands closest to the bullseye-marked reference point, which denotes a high Pearson Correlation Coefficient (PCC), low standard deviation, and low RMSE.

In comparison to the analyzed methods, Fig. 11 illustrates how the suggested framework attains a location that is closer to the reference point. This suggests:

- Greater Correlation: Our model’s PCC value indicates a more robust positive linear correlation between the expected and actual sales values, indicating that the model is successful in capturing the underlying trends in the sales data.
- Decreased Variability: Our model’s location on the diagram indicates a reduced standard deviation, which suggests that the forecasts are less erratic and more likely to match the real sales numbers.
- Reduced Prediction Errors: Our model’s closeness to the reference point visibly validates the reduced RMSE values shown in Fig. 6, which reinforces the model’s exceptional prediction accuracy.

## Discussion, limitations, and future directions

This section aims at identifying the role and relevance of the proposed framework and it’s fit with the existing literature for the enhancement of supply chain management in CBEC systems. After that, based on the findings of the present study, the limitations are pointed out and promising research avenues are proposed, followed by a discussion of the implications of the findings for CBEC enterprises.

### A. contribution to the existing literature

The proposed framework offers a novel approach to the management of supply chains in the context of CBECs by incorporating several crucial components. An attempt has been made to contribute to the existing literature in the following areas.

- Neural Networks for Demand Forecasting: The introduced approach to model complex nonlinear relations in the historical sales data includes the use of the neural network architecture, which provides more accurate demand forecasts in comparison with the methods SARIMA by Singh et al.^15^ and ARIMA-NARNN by Li et al.^17^.
- Black-Hole Optimization for ANN Training: In comparison with the approaches without optimization techniques (for instance, the CNN proposed by Pan et al.^16^), BHO ensures that the ANN will reach an optimal weight vector, which in turn, enhances the prediction precision.
- Fuzzy Logic for Resource Requirement Estimation: Compared to techniques that only use crisp values, we are able to better handle the vagueness of the sales data using fuzzy logic, which in turn provides a more accurate estimation of resources needed by the retailers.
- Auction Theory for Resource Allocation: The auction model provides a new solution to the problem left unaddressed in the literature by equally distributing the scarce supplier resources to retailers based on their expected demand.

### B. limitations and future research directions

Although the outcomes of the suggested framework are encouraging, there are several drawbacks to take into account as well as areas that warrant more study:

- Data Dependency: The accuracy of the model highly depends on the completeness and quality of the historical sales data. Some additional investigations in the future studies could be devoted to the use of more powerful techniques for handling missing data or merging different datasets.
- Factors from Outside the Model: The present model mainly uses the historical data of sales mostly from the past. Adding external features like competition, current economic climate, and sentiment analysis from social media might help enhance the predictability of results.
- Integration in real time: It is also possible that more dynamic and responsive choices are available for the allocation of resources when real-time sales data streams are incorporated into the model. To do this, though, would need solving potential problems in relation to computational effectiveness and data analysis.
- Expanding the criteria defined in the auction model: The decisions made in the presented auction model are restricted to the information on the current stock of resources and anticipated demand. It can be assumed that if additional criteria such as customer satisfaction indicators are taken into account in this auction model, it can become more realistic.

To strengthen and extend the proposed framework, it is possible to eliminate these shortcomings and focus on the directions for further research mentioned above. It will further improve the supply chain management optimization in the CBEC field which is changing dynamically.

### C. practical implications for CBEC businesses

Notwithstanding the mentioned drawbacks, the suggested framework provides CBEC enterprises with a number of benefits:

- Increased Demand Forecasting Accuracy: By providing more accurate predictions on the future demands, the proposed model makes it possible to manage resources more efficiently, regulate stocks and inventories, and launch the marketing strategies.
- Diminished goods Expenses: This implies that by adopting this method of demand estimation, firms are able to minimize on the costs of storing the excess inventories that are not in high demand in the market since the amount to be purchased is estimated accurately.
- Increased Level of Client Satisfaction: Demand forecasting enables the organization to have the right stock to attend to its customers’ needs and not to lose them due to delayed delivery of their orders.
- Optimized Resource Allocation: The auction-based resource allocation mechanism can be very effective in maximizing the overall sales within the CBEC system since it will ensure that the limited resources within the system are taken to the particular retailer who is expected to have highest demand for the product.

## Conclusion

Accurate sales forecasting, one of the key components of the success of e-commerce enterprises, can be accomplished by using hybrid information produced by prediction markets. Combining human analysts and machine learning algorithms is required. Additionally, this study suggests a brand-new approach to supply chain management optimization in cross-border e-commerce (CBEC) that combines fuzzy logic and auction theory to help make strategic choices that will increase productivity and profitability. The results of this article indicate our minimum error in RMSE and MAE values of 22.31 and 18.76, respectively, when compared to other comparison methodologies, demonstrating the superiority of our proposed method. As a result, managers are better equipped to make choices that improve the overall effectiveness of the supply chain at CBEC. In summary, the emergence of e-commerce has revolutionized the way businesses operate, and the utilization of hybrid intelligence and innovative strategies like the one proposed in this paper further contribute to the growth and success of e-commerce businesses by tackling the complexities and uncertainties of the digital market.

Although this study offers a viable strategy for CBEC supply chain optimization, it also identifies a number of areas in need of further development. Obtaining additional diverse data could help overcome the limits of the dataset, which include the number of retailers, duration, and product breadth. Due to the complexity of the model—especially the "black-box" nature of ANNs and possible computing cost—it is necessary to investigate optimization and interpretability solutions. Further thought must also be given to generalizability to various CBEC scenarios with differing platforms, suppliers, and dynamics. The study offers promising future directions. Important next stages include increasing data collection, implementing Explainable AI (XAI) for enhanced interpretability, and looking into techniques for increased efficiency and scalability. Further promising directions for expanding the impact of this research include investigating dynamic resource allocation, integrating multi-objective optimization, and guaranteeing smooth integration with current systems.

## Data Availability

All data generated or analysed during this study are included in this published article.
